# Wounding increases nuclear ploidy in wound-proximal epidermal cells of the *Drosophila* pupal notum

**DOI:** 10.17912/micropub.biology.001067

**Published:** 2024-03-02

**Authors:** James White, M. Shane Hutson, Andrea Page-McCaw

**Affiliations:** 1 Dept. Cell and Developmental Biology, Vanderbilt University, Nashville, Tennessee, United States; 2 Program in Developmental Biology, Vanderbilt University, Nashville, Tennessee, United States; 3 Dept. Physics and Astronomy, Vanderbilt University, Nashville, Tennessee, United States; 4 Dept. Biological Sciences, Vanderbilt University, Nashville, Tennessee, United States

## Abstract

After injury, tissues must replace cell mass and genome copy number. The mitotic cycle is one mechanism for replacement, but non-mitotic strategies have been observed in quiescent tissues to restore tissue ploidy after wounding. Here we report that nuclei of the mitotically capable *Drosophila* pupal notum enlarged following nearby laser ablation. Measuring DNA content, we determined that nuclei within 100 µm of a laser-wound increased their ploidy to ~8C, consistent with one extra S-phase. These data indicate non-mitotic repair strategies are not exclusively utilized by quiescent tissues and may be an underexplored wound repair strategy in mitotic tissues.

**Figure 1. Wounding affects the cell-cycle and ploidy in the Drosophila pupal notum:**
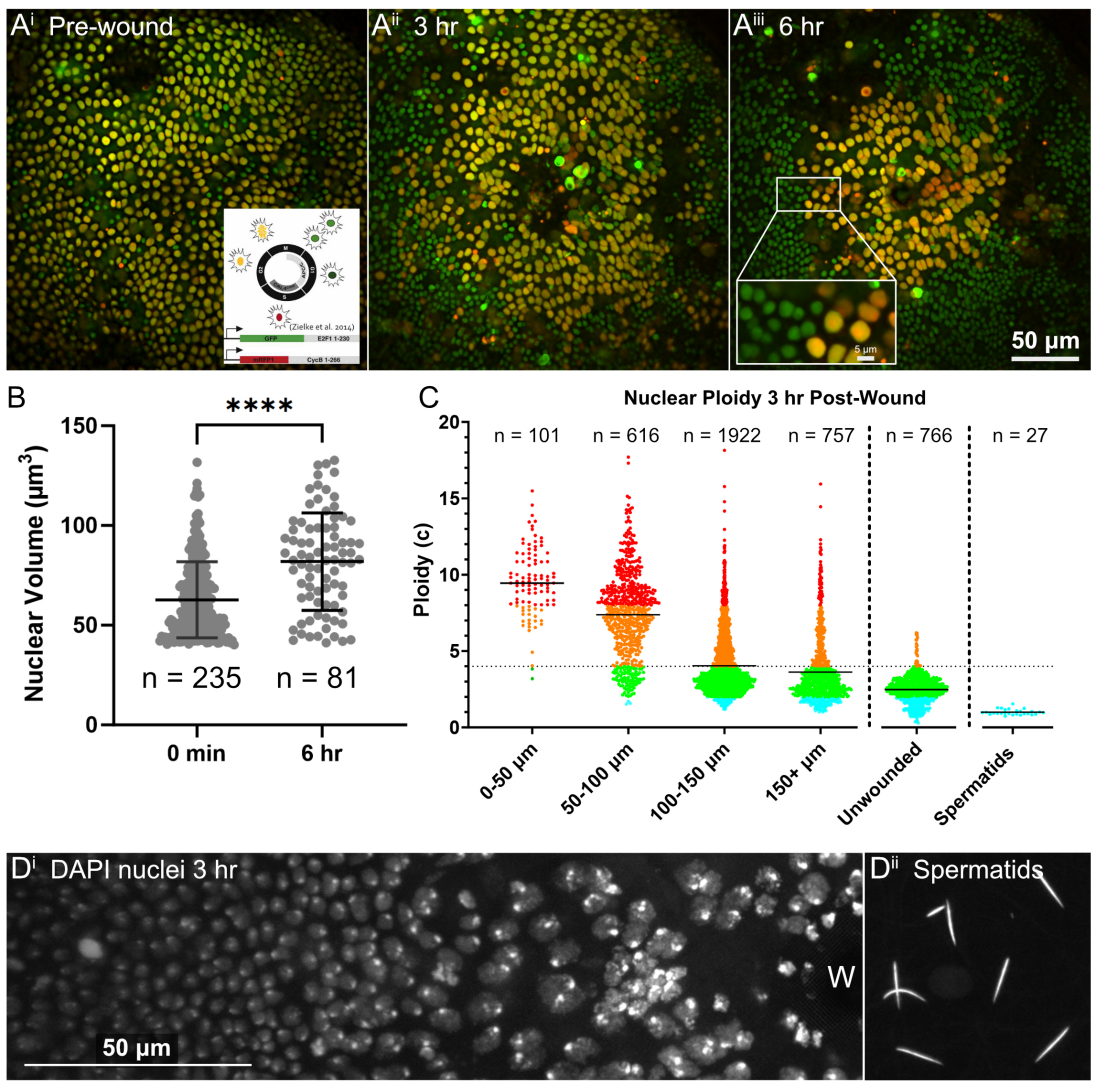
**A:** FUCCI fly indicating cell cycle state pre-wound, 3 hr, and 6 hr post wound in the *Drosophila* pupal notum (green nuclei = G1, red = S-phase, yellow = G2/M) Most nuclei are in G2 before wounding. Bar is 50 µm; inset bar in A ^iii^ is 5 µm. **B:** Nuclear volume of G2 (yellow) FUCCI nuclei segmented in 3D using NIS elements from 3 independent samples. Mean and standard deviation shown (black bars), Mann-Whitney test indicates p < 0.0001 (****) between 0 min and 6 hr post wounding. **C:** DNA content of nuclei at 3 h after wounding. DAPI intensity was measured in 3D-segmented nuclei then normalized to DAPI intensity in haploid spermatids. Each dot represents a nucleus, and data from 4 biological replicates (wounded nota) are combined. Results are binned into 50 µm distances from wound center with *n* total nuclei within each bin shown. For reference, ploidy was measured in an unwounded control sample. Solid lines indicate the mean of each category, dotted line indicated 4C cutoff. A one-way ANOVA with multiple comparisons comparing the unwounded control to distance bins resulted in p < 0.0001 to each bin. **D** : Nuclei increase in DNA content and size closer to the wound. **D ^i^** shows DAPI-stained nuclei 3 hr post-wounding. W=wound center. **D ^ii^** shows DAPI-stained spermatids used to normalize DNA content. Both images are max intensity projections, preprocessed with rolling ball correction before projection, 19 Z-slices in D ^i^ and 24 Z-slices in D ^ii^ . Bar is 50 µm and applies to D ^i^ and D ^ii^ .

## Description

Epithelia maintain barriers to the outside environment, but after epithelial injury the loss of barrier integrity allows pathogens to invade. To re-establish the barrier and restore homeostasis, cells must cover the wound area. The mitotic cell cycle replaces cell mass as well as genetic material, and mitosis is observed in epithelial cells near a wound in mouse skin (Park et al., 2017) . In the adult fly epidermis, however, epithelial cells are post-mitotic, and there is no stem cell pool to contribute new cells to close wounds. Previous studies have determined that in response to a wound, adult fly epidermal cells utilize endoreplication, which includes both growth (G) phases and S-phases but omits mitosis, resulting in larger cells with increased nuclear ploidy that close the wound (Losick et al., 2013; Losick et al., 2016) . These large polyploid cells not only endocycle but also fuse with each other to form syncytia (Besen-McNally et al., 2021; Grendler et al., 2019; Losick et al., 2013; Losick et al., 2016) . Similar non-mitotic repair strategies have been observed in other tissues (Cao et al., 2017; Edgar & Orr-Weaver, 2001; Gentric et al., 2015; González-Rosa et al., 2018; Lee et al., 2009; Orr-Weaver, 2015; Sigal et al., 1999; Wang et al., 2018; Wang et al., 2013; Wilkinson et al., 2019).

We have previously investigated wound responses in the *Drosophila* pupal notum (O'Connor et al., 2021; Shannon et al., 2017). Although this tissue is mitotic, we previously reported that epithelial cells near laser wounds fuse with their neighbors to form giant syncytia (White et al, 2023) . Here, to investigate the cell-cycle response to wounds, we laser-ablated *Drosophila* pupae expressing the Fluorescent Ubiquitination-based Cell Cycle Indicator (FUCCI). FUCCI is designed to provide a live readout of cell-cycle state where green fluorescence from GFP-E2F1 corresponds to a cell in G1, red fluorescence from CycB-RFP corresponds to a cell in S-phase, and both fluorophores are present in G2/M. The epithelial cells of the unwounded pupal notum undergo waves of mitosis (Guirao et al., 2015) ; we wounded at 12-15h APF, when most cells were in G2 (yellow), some are in M (fluorophores lost after nuclear envelope breakdown) and G1 (green), with very few in S phase (red) ( Fig. 1A ^i^ ). Post-wounding, wound-proximal nuclei did not progress through the cell cycle but rather maintained both fluorophores over the course of wound closure ( Fig. 1 A ^ii^ ,A ^iii^ ). Interestingly, at 6 h post wound, nuclei had an increased nuclear volume compared to immediately after wounding ( Fig. 1B ), suggesting these nuclei may be polyploid. To assess the ploidy of nuclei post-wound, we fixed and DAPI-stained the notum epithelium using a protocol we developed (White et al., 2022) , normalizing to haploid spermatids that were fixed and DAPI-stained along with the dissected notum (Fig.1 D ^i^ -D ^ii^ ). Within 3 h after wounding, nuclei within 100 µm from the wound averaged ~8C, consistent with one extra S-phase ( Fig. 1C ). In contrast, most nuclei further from the wound had ploidy levels within the diploid range, comparable to unwounded controls ( Fig. 1C ). Thus, even though the pupal notum epithelium is mitotic (Guirao et al., 2015; White et al., 2023) , laser ablation induces nuclear polyploidy as well as cell fusion.

Previous studies investigating developmental polyploid cells using the FUCCI system observed green (G1) or red (S) nuclei, as expected for endoreplication (Burbridge et al., 2021; Wang et al., 2021) ; thus, it is noteworthy that we observed a different signature of both fluorophores together. However, these previous studies analyzed unwounded tissues. In the context of injury, a previous study using FUCCI indicated G2 stalling followed injury in the diploid cells of the *Drosophila* wing imaginal disc (Cosolo et al., 2019) . It may be relevant that in our laser-ablation system, wound-proximal nuclei undergo significant damage after laser ablation (O’Connor et al., 2021). Most of these nuclei were in G2 when damaged, and they may not be able to execute mitosis in their damaged state, perhaps because they fail the G2/M checkpoint, or perhaps because the mechanical environment around the wound is not suited to mitosis (Han et al., 2023) . Thus, these cells may enter S-phase directly, resulting in a different FUCCI signature than developmental polyploid cells. We note that the G1 (green) marker is based on E2F1, usually destroyed during S phase; but in mammals DNA damage activates and stabilizes E2F1 (Lin et al., 2001; Wang et al., 2004; Zhang et al., 2009) , recruiting E2F1 to DNA damage sites (Choi & Kim, 2019) . The role of E2F1 in the DNA damage response of *Drosophila* , however, has not been explored.

Our results indicate that even a mitotically active tissue can induce nuclear polyploidy in response to damage. Why would a mitotic tissue do this? Note that these wounds are healed rapidly, within 3-6 hours, and we speculate that polyploidization may increase both cell mass and the amount of genetic material faster than the available mitotic cell cycle in a damage context.

## Methods

Flies :

Figure 1 A,B : *w ^1118^ ; Kr ^If-1^ /CyO, P{ry ^+t7.2^ =en1}wg ^en11^ ; P{w ^+mC^ =Ubi-GFP.E2f1.1-230}5 P{w ^+mC^ =Ubi-mRFP1.NLS.CycB.1-266}12/ TM6B, Tb ^1^* (Bloomington stock 55124)

Figure 1 C : *P{Ubi-p63E-shg.GFP}5 / CyO ; pnr-Gal4, UAS-mCherry.NLS, tubP-Gal80 ^ts^ / TM3* (Flybase unique identifiers: FBti0004011, FBti0151829, FBti0147460, FBti0027797)

Wounding :

Flies were mounted and wounded as described previously (O'Connor et al., 2022; White et al., 2023).

Live imaging the cell cycle :

Cell cycles were visualized with the Fluorescent Ubiquitination-based Cell Cycle Indicator (FUCCI) fly (Zielke et al., 2014) . Images were captured pre-wounding, immediately post-wound, and every ten minutes on a Nikon Ti2 Eclipse with X-light V2 spinning disc (Nikon, Tokyo, Japan) using a 60x oil-immersion objective.

Volume of FUCCI nuclei:

FUCCI nuclei with both E2F1-GFP and CycB-RFP were segmented in 3D using NIS Elements GA3. Brightspots segmentations were made for both E2F1-GFP and CycB-RFP and only nuclei that contained both fluorophores were analyzed to exclude the smaller G1 green-only nuclei. Volume measurements were performed both immediately after wounding and 6 h post-wound, exported to excel, and graphs were generated using GraphPad Prism 10.

DAPI staining and measurement :

*ShgGFP / CyO ; PnrGal4, Gal80 ^ts^ , UAS-nuc-mCherry / TM3* pupae were mounted, wounded, and allowed to recover for 3 h. Spermatids were dissected from healthy unwounded male flies and allowed to dry on a 24x60 mm coverslip. Two wounded pupae were isolated on the coverslip with dried spermatids, then dissected and fixed as previously described (O'Connor et al., 2022; White et al., 2022). Pelts and spermatids were incubated with 1 µg/ml DAPI for 45 min to allow the stain to completely penetrate the tissue, then washed and mounted as described previously (White et al., 2022) .

DAPI intensity was assessed by creating an NIS elements macro to segment epithelial nuclei in a 3D volume. To create the segmentation macro, we used NIS elements General Analysis 3. Nuclei were segmented in 3D based on a nuclear-localizing mCherry fluorophore. mCherry signal was enhanced with the Local Contrast preprocessing and then segmented with Brightspots detection. Single voxel Brightspots were grown to fill the whole mCherry labeled nucleus. Nuclear segmentations were then filtered based on volume to exclude erroneously small and large objects, as well as sphericity to remove erroneously fused objects. Subtle variance in mCherry signal intensity across nuclei meant a single Brightspots setting would miss many nuclei. So, three Brightspots detections were run in parallel each with subtly different thresholding parameters to capture most nuclei. Outputs of the three Brightspots segmentations were merged into one binary image which was used to obtain DAPI intensity within nuclei. Some areas of DAPI stain were contaminated by an underlying bright muscle band signal. So, the DAPI channel was thresholded by eye for each sample so only nuclei within uncontaminated areas were included. Basal immune and blood cell nuclei are not entirely removed during dissection. To exclude these nuclei, only nuclei near the apical epithelial border marker E-cadherin GFP (ShgGFP) were analyzed. E-Cad GFP signal was refined with rolling ball and local contrast pre-processing and then thresholded to create a binary E-Cad sheet. The threshold was filtered by volume to remove small artifacts not connected to the E-Cad tissue sheet. The E-Cad Sheet was then dilated and only nuclei that fell within the dilated E-Cad sheet were analyzed. Nuclei were then assigned a distance from the wound bed by thresholding the dim E-Cad signal within the wound bed, which was refined by erosion and filtering by volume to leave only a single wound bed object. All nuclei were then assigned a distance from the center of the wound bed object. Finally, the sum intensity of the DAPI signal was determined for all nuclei collated with the distance from the wound and then output to a CSV file.

A 1C (haploid) standard was created by imaging spermatids on the slide with the same conditions as the pupae. Spermatids were thresholded and filtered by volume and elongation to remove erroneous objects. Sum DAPI intensity was taken for all filtered spermatids and exported as a CSV. Within the CSV, the average intensity of spermatids was determined, corresponding to a haploid genome or 1C. All nuclei intensity values were divided by the average spermatid value resulting in the ploidy in C for nuclei and their distance from the wound. Within the CSV nuclei were sorted by distance from the wound and exported to prism.
